# Permethrin Susceptibility for the Vector* Culex tarsalis* and a Nuisance Mosquito* Aedes vexans* in an Area Endemic for West Nile Virus

**DOI:** 10.1155/2018/2014764

**Published:** 2018-07-11

**Authors:** Geoffrey P. Vincent, Justin K. Davis, Michael C. Wimberly, Christopher D. Carlson, Michael B. Hildreth

**Affiliations:** ^1^Department of Biology and Microbiology, South Dakota State University, Brookings, SD 57007, USA; ^2^Geospatial Sciences Center of Excellence, South Dakota State University, Brookings, SD 57007, USA; ^3^South Dakota Department of Health, Public Health Laboratory, Pierre, SD 57501, USA; ^4^Department of Veterinary and Biomedical Sciences, South Dakota State University, Brookings, SD 57007, USA

## Abstract

In 2016, we compared susceptibility to the insecticide, permethrin, between the West Nile virus vector,* Culex tarsalis* Coquillett, and a major nuisance mosquito,* Aedes vexans* (Meigen), using baseline diagnostic dose and time values determined using the CDC bottle bioassay protocol. Mosquitoes were collected in the wild in Brookings County, South Dakota, situated in the Northern Great Plains of the USA. The determined diagnostic dose and time were then used in 2017 to validate these measurements for the same 2 mosquito species, collected at a second location within Brookings County. The diagnostic dose was determined for multiple time periods and ranged from 27.0 *µ*g/ml at 60 min to 38.4 *µ*g/ml at 30 min. There was no significant difference detected in mortality rates between* Cx. tarsalis* and* Ae. vexans* for any diagnostic time and dose. For practical purposes, mosquitoes in 2017 were tested at 38 *µ*g/ml for 30 min; expected mortality rates were 93.38% for* Cx. tarsalis* and 94.93% for* Ae. vexans.* Actual 2017 mortality rates were 92.68% for* Cx. tarsalis* and 96.12% for* Ae. vexans*, validating the usefulness of this baseline at an additional location and year.

## 1. Introduction

Mosquito transmission of arboviruses to humans depends on multiple factors [1, 2], including human behavior [3, 4]. Gujral et al. [3] suggest that human behavioral risk factors, such as the use of personal protective measures, can be influenced by the “biting pressure” created by local mosquito populations. These populations include potential vector species and nonvector nuisance mosquitoes, and the presence of many nuisance mosquitoes could increase the use of personal protectants or avoidance behavior, thereby reducing the chance of potential viral transmission by vector mosquitoes. Conversely, the lack of abundant nuisance mosquitoes may have the opposite effect. Therefore, it is important to consider nuisance as well as vector mosquitoes when developing comprehensive strategies for mosquito reduction and disease control.

Community adulticiding efforts can limit both disease and nuisance issues caused by mosquitoes, but the common use of insecticides has prompted concerns over growing resistance to insecticides in mosquito populations. Permethrin, a broad-spectrum insecticide in the pyrethroid family, is the primary adulticide used in the United States [5] and is used for agriculture to reduce crop and livestock pests [6, 7], as well as in residential areas to control nuisance and vector mosquito populations. Long-term usage of this class of insecticide may cause increased resistance in mosquito populations [8]. Because of its broad use and the documented cases of resistance, monitoring of permethrin resistance is important to mosquito control efforts [9, 10].

Most studies on insecticide resistance have focused primarily on vector mosquito species, and few have included common nuisance mosquitoes within arbovirus-endemic areas. Richards et al. (2017) evaluated the susceptibilities of a potential Zika virus (ZIKV) vector (*Aedes albopictus* (Skuse)) and 2 West Nile virus (WNV) vectors (*Culex pipiens* L. and* Culex quinquefasciatus* (Say)) to 6 different common insecticides in a study that included 26 mosquito populations from four different USA regions. They also included the tree-hole mosquito,* Aedes triseriatus* (Say), in this study, though this species is not a significant vector for Zika or WNV and is generally only a minor species in most regions. They found that the* Aedes* species tested were less likely to exhibit resistance when compared with* Culex* species, particularly for etofenprox and malathion, and found that all* Aedes* spp. populations tested were either susceptible or possibly resistant to permethrin while most* Culex *spp. populations were resistant. Given the potential role of nuisance mosquitoes in encouraging the use of personal protectants, reduction in their populations during high-risk transmission periods could lead to a reduced perceived threat of mosquito-borne diseases by the general [4] which could increase exposure to pathogens. Therefore, the susceptibility of nonvector mosquitoes to insecticides should be evaluated especially in arbovirus-endemic regions where nuisance species are far more abundant than the vector species.

In the USA Northern Great Plains,* Culex tarsalis *Coquillett is the primary vector for WNV, and* Aedes vexans* (Meigen) is generally the most predominant nuisance mosquito [11–14]. Recently,* Ae. vexans* has been reported as a potential vector for ZIKV [15, 16]; however, there have been no reported cases of local transmission of ZIKV in the Northern Great Plains. In eastern South Dakota,* Ae. vexans* populations generally swell to very large numbers in the spring and remain high during the WNV transmission season [17]. The aggressive biting of this nuisance mosquito can motivate people to use personal protection [3, 4]. In spite of nuisance and potential public health significance of each species, we have found no studies directly comparing permethrin susceptibility for* Cx. tarsalis *and* Ae. vexans*.

Brookings County, located in east-central South Dakota, is the fifth-most populated county and contains the fourth largest city in South Dakota, though it mostly consists of farmland. Both species of interest are abundant in this county with* Ae. vexans *having the largest abundance of all mosquito species. The city of Brookings has utilized a mosquito control program targeting both nuisance and vector mosquitoes, which included larviciding potential habitats and general spraying of permethrin for over 20 years, and the small cities in the county have had similar programs for over 10 years. The purpose of the present study is to compare susceptibilities of* Ae. vexans* and* Cx. tarsalis* to reagent-grade permethrin in a Center for Disease Control (CDC) bottle bioassay protocol involving multiple concentrations and time periods. For this comparison, we used adult mosquitoes freshly captured in Brookings County, an endemic area for WNV, using CO_2 _ baited light traps. Because both species prefer to lay their eggs throughout natural habitats, harvesting and rearing* Cx. tarsalis* and* Ae. vexans *eggs for the assay were not practical, and results can be inconsistent when rearing mosquitoes from eggs in a lab [10]. In our area, the consistent collection of* Cx. tarsalis *larvae in large enough numbers to adequately compare its susceptibility to* Ae. vexans *was also not practical. To minimize concerns about potential high variability for data collected from wild-caught adults, the susceptibility comparisons involved a large number of mosquitoes evaluated in multiple assays conducted throughout the mosquito season. The use of field-collected mosquitoes for this comparison also allowed for testing both species together in the same bottles and testing them in the various natural physiological conditions representative of the wild populations' age distribution at any given time [18].

The data from this study were used to calculate a baseline diagnostic dose and time for permethrin susceptibility for both species that can be used in future studies for evaluating changes in permethrin resistance from this region. We have found no studies identifying values for diagnostic dose and time on permethrin susceptibility for* Cx. tarsalis *and* Ae. vexans*, and none have been reported to the Arthropod Pesticide Resistance Database [19]. According to the CDC diagnostic dose and times should be determined for each type of insecticide used and for each species of concern within a region [20], and studies often emphasize the lack of reported diagnostic dose and times reported for* Culex *species, and more so with* Cx. tarsalis* [21]. CDC guidelines recommend creating the diagnostic dose and time from susceptible populations; however, permethrin is used throughout the state for both agricultural and mosquito control purposes. The Center for Disease Control guidelines also recommend establishing these baselines from mosquito populations in areas where treatments are applied as a reference point for future comparisons.

## 2. Methods

### 2.1. Mosquito Collections

From June through August of 2016, mosquitoes were captured overnight using one CDC Miniature Light Trap Model 512 equipped with photoswitches and air-actuated gate system (John W. Hock Company, Gainesville, Florida) and baited with CO_2_ at a farm located 1.8 km from the southwestern city limits of Brookings, South Dakota (44.25°, -96.81°). Mosquitoes were collected each morning shortly after dawn. This site was selected as populations of* Cx. tarsalis* and* Ae. vexans* are present in large enough abundances to test mortality rates for multiple permethrin concentrations. This location is also on the outskirts of a city where insecticide fogging applications targeting mosquitoes have occurred sporadically for 25 years. In June of 2017, mosquitoes were again collected using two CDC Miniature Light Traps from Oakwood State Park (44.45°, -96.98°) located 25 km northwest. In neither year were targeted mosquito control applications or agricultural applications of insecticide administered near either site. Mosquito species were identified based upon morphological characteristics [22].

### 2.2. CDC Bottle Bioassay

The CDC bottle bioassay was chosen for its effectiveness in testing field-collected mosquitoes [23, 24]. Calibration of the CDC bottle bioassay was performed per the CDC bottle bioassay protocol [20]. These guidelines recommend that a baseline diagnostic value should be determined for use in comparisons for future resistance testing on specific mosquito species and geographical regions. In determining the diagnostic dose and times, we identified a concentration that would result in 100% mortality between 30 and 60 min and then estimated the LC98, or the lowest concentration which was lethal for 98% of mosquitoes at those time spans. For the 2016 bioassay calibration, tested concentrations were 1, 10, 20, and 40 *µ*g/ml of laboratory grade permethrin (Sigma-Aldrich, St. Louis, Mo.) suspended in acetone. Due to varying physiological states of wild-caught mosquitoes, replicates of each concentration were run until at least 100 mosquitoes of each species were tested, which ranged from 9 to 24 replicates depending on the availability of species at the time of the trial. Additionally, any mosquitoes that died or had physical injuries at the start of each trial, such as broken or missing wings or legs, were excluded. All trials included four treatment bottles and one negative control in which the bottle was coated with acetone only. Each test bottle was cleaned with commercial-grade detergent, rinsed thoroughly three times with tap water, and then dried in an oven at 50°C for 20 minutes to ensure removal of residual contaminants per the CDC bottle bioassay protocol. Control bottles were selected randomly to further ensure bottle cleaning was thorough. Each treatment consisted of clear 250 ml bottles coated with 1 ml of a specific concentration containing permethrin with 1 control bottle coated with 1 ml of acetone only. For the 2017 validation bioassays, permethrin was prepared at a concentration of 38 *µ*g/ml based upon the results from the 2016 calibration assay.

Prior to testing, captured mosquitoes were maintained in 30.5 cm X 30.5 cm X 30.5 cm holding cages in the laboratory for 3 to 4 hours to acclimate to the environment of 23°C and 50% relative humidity and then removed any mosquitoes that may have died during the trapping process. Mosquitoes were transferred to the experimental bottles via a mechanical aspirator to avoid introducing condensation to the treatment bottles. The CDC bottle bioassay protocol recommended determining diagnostic times between 30 and 60 minutes so mosquito mortality was measured every 5 min for 60 min. Mosquitoes that would no longer right themselves after slowly rolling the bottle were considered dead. Mosquitoes that survived past 60 min were removed from the bottle and euthanized separately. During the 2017 bioassays, mosquitoes were only evaluated for 30 min. Afterwards, all mosquitoes were identified to species. Only data from* Cx. tarsalis* and* Ae. vexans* were used in this study. Corrected mortality was not needed as no mortality occurred in any of the control bottles.

### 2.3. Statistical Analyses

Using mosquitoes collected in 2016, a probit model (glm function, stats package, R x64 version 3.2.2) was used to model the relationship between proportion mortality (dead per total in bottle, binomial family) as a linear function of time, with slope and intercept depending on species as a factor and concentration linearly. The estimated diagnostic dose or LC98 was defined as the lowest dose for which the expected mortality exceeded the susceptibility threshold defined by the CDC (98% mortality) for each species and concentration at 30, 45, and 60 min [18, 20].

A likelihood ratio test (ANOVA function, stats package) was performed against a simpler model in which mortality depended on concentration and time but not species, to determine whether the 2 species differed by mortality in any systematic way during the experiment. For analysis of the 2017 mosquito data, 95% exact confidence intervals were calculated for proportion mortality at the chosen dose and time (binconf function, Hmisc package) to compare with estimates obtained in 2016.

## 3. Results

**Figure 1:**
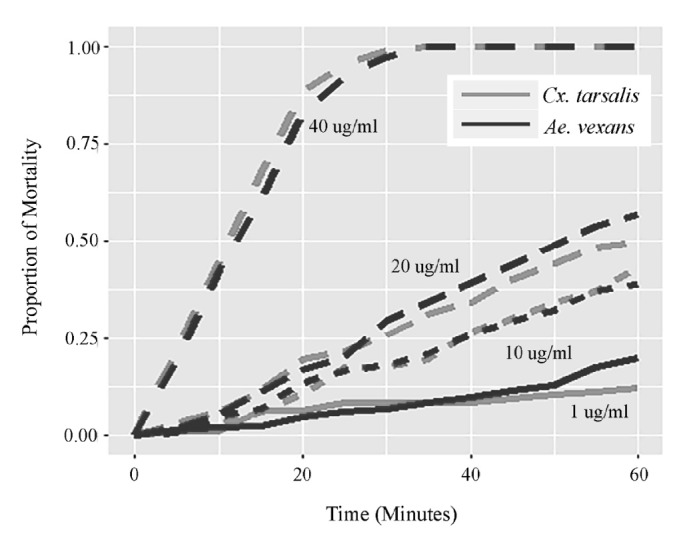
Mortality rates for* Cx. tarsalis* and* Ae. vexans* as a function of time for various concentrations of permethrin in 2016 using probit model.

*Culex tarsalis* (n = 421) and* Ae. vexans* (n = 1084) were tested in 2016 at various concentrations of permethrin. An additional 159* Cx. tarsalis* and 207* Ae. vexans* were used in control bottles containing acetone only. No mortality in the control bottles occurred during the experimental period. Observed mortality rates as functions of time, species, and permethrin concentration are displayed in Figure 1. Mortality rates increased as permethrin concentration increased and were nearly linear in time for each concentration of permethrin except for 40 *µ*g/ml, which reached near 100% mortality at approximately 30 min for both species. At 20 *µ*g/ml, mortality reached 50% for both target species at 60 min.

**Figure 2:**
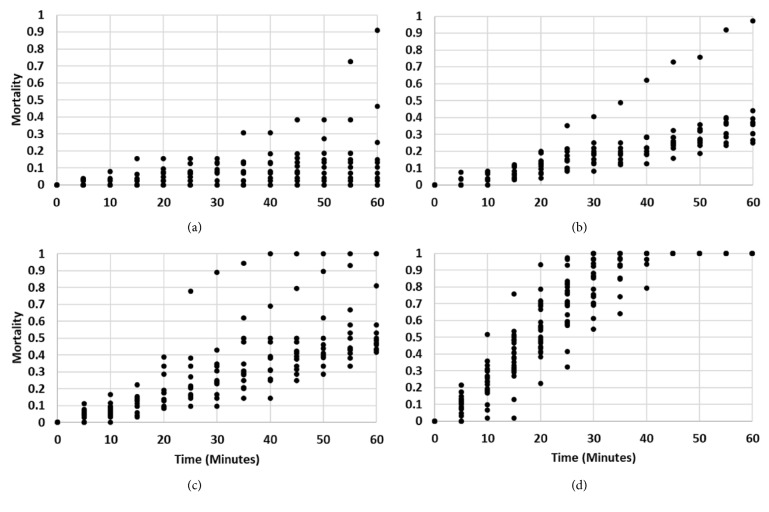
Observed mortality rates for each bottle bioassay replicate in 2016 at time intervals for 1 ug/ml (a), 10 ug/ml (b), 20 ug/ml (c), and 40 ug/ml (d) of permethrin.

The estimated LC98 for* Ae. vexans* was 38.4 *µ*g/ml at 30 min and 27.0 for 60 min. For* Cx. tarsalis*, it was 38.3 ug/ml at 30 min and 27.5 *µ*g/ml at 60 min (Table 1). This showed that increased permethrin concentrations achieved the same mortality in less time for both species. For a given time, mortality rates are similar between the 2 species. We saw some variation after 50 minutes at 1 ug/ml where mortality rates in* Ae. vexans* were higher than* Cx. tarsalis*, but this may have been due to one trial rapidly spiking to 70% mortality near 55 minutes (Figures 1 and 2(a)). Mortality rates at 10 ug/ml were consistent for both species at most times with one trial reaching near 100% mortality at 60 minutes (Figures 1 and 2(b)).* Aedes vexans* showed a slightly higher mortality rate at 20 ug/ml for between 30- and 60-minute time intervals (Figure 1) with 1 trial reaching 100% mortality at 40 minutes and a second at 60 minutes (Figure 2(c)). Mortality rates at 40 ug/ml for* Cx. tarsalis* were slightly higher than* Ae. vexans* at earlier times; however, both species reached 100% mortality at approximately the same time interval (Figure 1). Mortality rates reached 100% in all trials within the bioassay time limit. Using a diagnostic time longer than 30 min is likely inadvisable, as both species showed 100% mortality soon after that for the 40 *µ*g/ml concentration (Figure 1).

A likelihood ratio test between the main model, used to calculate LC98s, and a simplified model, in which species was no longer a predictor, indicated that the mortality rates of the 2 species had statistically indistinguishable rates of mortality over concentration and time (p = 0.7803), and any function of model estimates (such as the diagnostic dose for a given time) is unlikely to differ between the 2 species.

**Figure 3:**
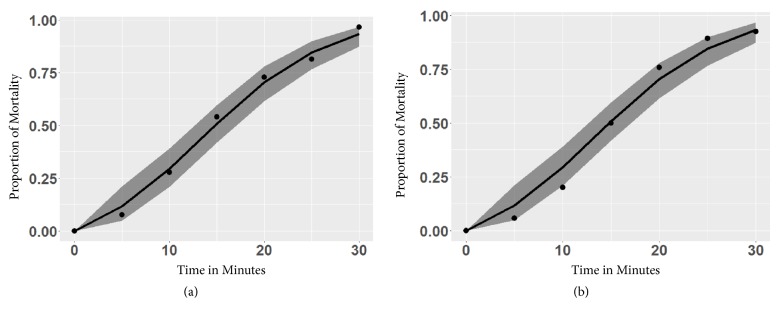
Bioassay mortality rates for* Ae. vexans* (a) and* Cx. tarsalis* (b) collected in 2017 (solid circles) using 38 *µ*g/ml of permethrin, against estimated calibration curve (line) with 95% confidence interval (grey shaded band) generated from data collected in 2016.

During the 2017 study, the estimated diagnostic doses determined in 2016 were rounded to 38 *µ*g/ml for both species at 30 minutes. This dose was chosen rather than the estimated LC98s both for convenience and also at least some (approximately 1 in 20) mosquitoes should be alive at 30 min. The mortality rates based on our model's diagnostic dose estimated mortality at 30 minutes to be 94.93% for* Ae. vexans* and 93.38% for* Cx. tarsalis*. In early 2017, 380* Ae. vexans* and 123* Cx. tarsalis* adult females were used to validate these calculations. Observed mortality rates for mosquitoes at the diagnostic time for* Ae. vexans* populations collected in 2017 at Oakwood State Park were 297/309 = 96.12% with a 95% exact confidence interval of 93.31 to 97.98%. Mortality rates for* Cx. tarsalis* collected at Oakwood State Park at the diagnostic time were 114/123 = 92.68% with 95% exact CI (86.56, 96.60%). In all cases, the 95% CI for observed mortality in 2017 contained the expected mortality rate estimated in 2016. For both species, estimated and observed mortality rates as functions of time are displayed in Figure 3.

## 4. Discussion

This study is the first direct comparison of the permethrin susceptibilities of a major vector for WNV,* Cx. tarsalis*, to a major nuisance mosquito,* Ae. vexans*, where WNV is endemic to the region, and was conducted using collected adult mosquito from eastern South Dakota exposed to multiple concentrations of permethrin in a CDC bottle bioassay. Scatter-plots of the data showed that while there were some variations in individual bottles, there was good consistency in bottles for each permethrin concentration (Figure 2). The overall results showed similar mortality rates at all times for all concentrations between* Cx. tarsalis* and* Ae. vexans*, and we found no statistical difference between the calculated mortality rates of these 2 species. Comparisons were made based upon calculated diagnostic dose and times as recommended by CDC when testing for insecticide resistance [17], and the similarities between each species were also reflected in the calculated diagnostic doses and times. The susceptibilities of* Ae. vexans* and* Cx. tarsalis* remained constant between 2016 and 2017, between 2 study sites, and within and between both species. A California study used 30 *µ*g/ml of permethrin and was unable to knock down 50% of their wild-caught* Cx. pipiens* [25]. Richards et al. [21] found 2* Aedes* spp. collected from 13 different locations within the United States that were either susceptible or possibly resistant with mortality rates ranging from 91% to 100% using 15 *µ*g/ml doses at 30 min. This same study tested 26* Cx. pipiens* collected from St. Paul, Minnesota, and achieved 96% mortality using 15 *µ*g/ml permethrin at 30 min. A similar study to ours tested 49* Cx. tarsalis* adults reared from field caught larva and determined a median lethal dose of 50 *µ*g/ml; however, they reported that this same concentration caused 100% mortality the following year and had to reduce their median lethal dose to 10 *µ*g/ml [10]. A diagnostic dose and time for* Ae. vexans* have not been reported; however, the CDC reports that 100% mortality should occur at 30 minutes for* Aedes* spp. at 15 ug/ml [26]. Our study at 20 ug/ml only resulted in 25% mortality at 30 minutes for* Ae. vexans* and our calculated value for 98% mortality was 38 *µ*g/ml in 30 min, over twice that of the CDC suggested guidelines. This same CDC report suggests a baseline diagnostic dose for* Cx. tarsalis* as 43 ug/ml at 30 minutes. Our calculated diagnostic dose and time for C*ulex tarsalis* were lower than the CDC recommendation and at 38.3 ug/ml at 30 minutes. These values indicate that* Ae. vexans* in this region have become resistant to permethrin at the same level of susceptibility as* Cx. tarsalis.*

Use of wild-caught adult mosquitoes in this type of bioassay is considered acceptable from both the CDC bottle bioassay protocol and the WHO test procedures for insecticide resistance, and wild-caught adult mosquitoes have been used in previous studies where mosquito aquatic stages were not consistently available [18, 20, 27, 28]. Our trials were able to attain consistent results from mosquitoes with varying physiological attributes between years and geographical locations, and the results should capture the variation found in the wild. This is essential in disease control and mosquito abatement programs whose primary concern is to reduce vector and/or nuisance populations [29]. Further studies investigate variations in insecticide effectiveness between age and physiological statuses. We determined multiple options for baseline diagnostic doses and times that can be used in the Northern Great Plains. By using this method, we have a basis for comparing various species levels of susceptibility and it allows for future testing and comparisons of resistance in and between* Cx. tarsalis* and* Ae. vexans* using the CDC bottle bioassay.

Both the vector and nuisance mosquitoes are heavily targeted within the state of South Dakota due to the former's ability to transmit WNV and the latter's overwhelmingly large abundance creating a nuisance for residents. Since nuisance mosquitoes may help motivate humans to seek shelter or use personal protection [3, 4, 30], higher susceptibility to insecticides in nuisance mosquitoes compared to vector mosquitoes may cause mosquito control efforts to actually increase human risk for WNV. Our findings showed that the susceptibility to permethrin between the WNV vector,* Cx. tarsalis,* and the nuisance mosquito,* Ae. vexans*, was similar in this region and that treatment will be equally effective; thus it will not diminish the nuisance mosquito more than the vector. However, the development of resistance in* Ae. vexans* should be of concern in that the control of the most abundant mosquito in the state is less effective than it should be. Considerations should be made to find more effective insecticides to control this nuisance mosquito. There is a great need for future studies to understand the level of insecticide resistance developing in vector mosquitoes throughout the United States; however, these studies should also include predominant nuisance mosquitoes in areas where both groups are abundant to ensure that susceptibility of the vectors is similar or higher so that attempts to control vector species will not have a significantly greater effect on nuisance mosquitoes, thus lowering the avoidance behaviors in humans.

## Figures and Tables

**Table 1:** LC98 diagnostic doses and times for *Cx. tarsalis* and *Ae. vexans* with lower (LCL) and upper (UCL) confidence limits.

Species	Time (min)	Diagnostic dose (ug/ml)	LCL (ug/ml)	UCL (ug/ml)
*Cx. tarsalis*	30	38.3	36.9	40.0
*Ae. vexans*	30	38.4	37.0	40.0
*Cx. tarsalis*	45	32.1	30.8	33.6
*Ae. vexans*	45	31.8	30.5	33.3
*Cx. tarsalis*	60	27.5	26.3	28.8
*Ae. vexans*	60	27.0	25.9	28.3

## Data Availability

The datasets created and analyzed during the current study are available from the corresponding author on reasonable request.
